# Aging, Protein Aggregation, Chaperones, and Neurodegenerative Disorders: Mechanisms of Coupling and Therapeutic Opportunities

**DOI:** 10.5041/RMMJ.10088

**Published:** 2012-10-31

**Authors:** Ehud Cohen

**Affiliations:** Biochemistry and Molecular Biology, The Institute of Medical Research Israel-Canada, The Hebrew University of Jerusalem—Hadassah Medical School, Ein Karem, Jerusalem, Israel

**Keywords:** Aging, insulin/IGF signaling, proteostasis, Alzheimer’s disease

## Abstract

Late onset is a key unifying feature of human neurodegenerative maladies such as Alzheimer’s and Parkinson’s diseases and prion disorders. While sporadic cases typically emerge during the patient’s seventh decade of life or later, mutation-linked, familial cases manifest during the fifth or sixth decade. This common temporal emergence pattern raises the prospect that slowing aging may prevent the accumulation of toxic protein aggregates that lead to the development of these disorders, postpone the onset of these maladies, and alleviate their symptoms once emerged. Invertebrate-based studies indicated that reducing the activity of insulin/IGF signaling (IIS), a prominent aging regulatory pathway, protects from neurodegeneration-linked toxic protein aggregation. The validity of this approach has been tested and confirmed in mammals as reducing the activity of the IGF-1 signaling pathway-protected Alzheimer’s model mice from the behavioral and biochemical impairments associated with the disease. Here I review the recent advances in the field, describe the known mechanistic links between toxic protein aggregation and the aging process, and delineate the future therapeutic potential of IIS reduction as a treatment for various neurodegenerative disorders.

## PROTEOSTASIS

A series of folding events and post-translational modifications are required for the proper maturation of nascent polypeptides. Specialized biological mechanisms act in the endoplasmic reticulum and in the cytosol to assist newly synthesized molecules to attain their desired, functional spatial structure.^1^ Similarly, mature proteins are under constant quality control surveillance aimed to identify unfolded and damaged molecules. Polypeptides that failed to fold properly and damaged mature proteins are designated for degradation by one of the cellular degradation mechanisms, the ubiquitin proteasome system (UPS)^2^ or autophagy.^3^ The clearance of these molecules avoids deleterious effects associated with protein misfolding and aggregation (proteotoxicity) and enables the recycling of amino acids. These co-ordinated cellular activities of protein folding, post-translational modification, quality control, and degradation maintain a functional protein network and were termed “proteostasis.”^4^ Despite the activity of the proteostasis network, subsets of aggregation-prone proteins fail to fold properly, escape degradation, and form insoluble aggregates that accumulate within the cell. In some cases this aberrant protein aggregation leads to the development of maladies that were collectively termed “conformational diseases.”^5^ The late-onset neurodegenerative maladies such as Alzheimer’s and Parkinson’s diseases, amyotrophic lateral sclerosis (ALS), and prion disorders are a prominent type of conformational diseases.

## NEURODEGENERATIVE DISORDERS STEM FROM ABERRANT PROTEIN AGGREGATION

Alzheimer’s disease (AD), the most prevalent neurodegenerative disorder, stems from a dual digestion of the amyloid precursor protein (APP) by two proteases, β and γ secretases, which release the Aβ family of aggregation-prone peptides (collectively referred to as “Aβ”). Due to its hydrophobic nature, Aβ rapidly forms aggregates of various sizes.^6^ Small Aβ aggregative structures (also known as “oligomers”) have been shown to be the most toxic species and to correlate best with the development of AD which is characterized by neuronal loss, neuro-inflammation, cognitive failure, and eventually death.^7^,^8^ Hitherto, the mechanistic details of how Aβ oligomers lead to the manifestation of AD are poorly understood. Mutations that increase the production of Aβ have been shown to increase the risk to develop familial AD,^9^ and a mutation that reduces the Aβ production was found to be protective.^10^ Yet, it is important to note that not all AD-associated mutations in presenilin-1, the active component of the γ secretase complex, lead to increased Aβ production suggesting that more than one mechanism underlie the development of AD.^11^ Indeed, presenilin-1 was also reported to have roles in autophagy-mediated protein degradation.^12^,^13^ In addition, AD is characterized by the formation of intra-cellular tangles of hyper-phosphorylated TAU, a microtubule-associated protein. The links of these tangles to the etiology of AD are largely obscure.^6^

Parkinson’s disease (PD) is a common movement disorder that emerges as a result of aberrant aggregation of the protein α-synuclein. This aggregation process decimates the dopaminergic neurons of the substantia nigra, resulting in various phenotypes including tremor, rigidity, and impaired movement.^14^ Similarly, the aggregation of mutant huntingtin, bearing an abnormally long polyglutamine stretch (polyQ), causes Huntington disease (HD).^15^

Most cases of AD and PD manifest sporadically during the seventh decade of life or later, while the rarer familial, mutation-linked cases appear during the patient’s fifth or sixth decade of life. The common temporal emergence pattern of different neurodegenerative maladies defines aging as the major risk factor for the development of these disorders.^16^ One possible explanation for the observation that diseases which exhibit different etiologies and cell biological features onset in surprisingly similar temporal emergence patterns suggests that the aging process plays an active role in enabling neurodegenerative maladies to onset late in life. This model suggests that protective mechanisms that prevent neurodegenerative disorders from emerging early in life are negatively regulated by aging, a process that exposes the aged organism to proteotoxicity and disease. The exploration of aging-regulating pathways during the last two decades enabled a comprehensive evaluation of this hypothesis.

## AGING IS A HIGHLY REGULATED PROCESS

Three independent mechanisms have been found to regulate aging and lifespan of model organisms: dietary restriction,^17^,^18^ the mitochondrial electron transport chain,^19^–^21^ and the insulin/IGF signaling (IIS) pathway.^22^,^23^ Among these, the IIS is the most prominent and best characterized mechanism whose knock-down possesses the most prominent effect on lifespan of flies, worms, and mice.^22^ In the nematode *Caenorhabditis elegans* (*C. elegans*) DAF-2, the sole insulin/IGF receptor, initiates a signaling cascade that negatively regulates its downstream transcription factors, DAF-16/FOXO, SKN-1/NRF, and the heat-shock factor 1 (HSF-1). The IIS activates a set of kinases that directly phosphorylate DAF-16^24^ and SKN-1^25^ to prevent their entry to the nucleus. Similarly, the IIS negatively regulates HSF-1 by preventing the phosphorylation of DDL-1, an HSF-1-interacting protein that when not phosphorylated retains this transcription factor in the cytosol.^26^ Thus, IIS reduction by *daf-2* knock-down hyper-activates its downstream transcription factors creating youthful, long-lived, stress-resistant worms.^22^ Since both DAF-16 and HSF-1 are known to be regulators of several genes encoding heat-shock proteins,^27^,^28^ it is plausible that these transcription factors promote longevity via the maintenance of proper protein homeostasis in late stages of life.^4^

Several studies in mouse models have indicated that the role of the IIS as a lifespan and aging regulator is highly conserved from worms to mammals. First, knocking down one copy of the mouse IGF-1 receptor (*Igf1r*), the closest *daf-2* orthologue in mammals,^29^ results in longevity and elevated oxidative stress resistance of the animals compared to their litter-mates which carry two *Igf1r* copies.^30^ Similarly, the knock-out of the insulin receptor in the adipose tissues of mice (FIRKO mice) leads to extended longevity,^31^ and mice lacking the insulin receptor substrate 1 (IRS1) are also long-lived.^32^

The findings that the regulation of aging by the insulin and IGF-1 signaling pathways are conserved in the mouse raised the question of whether these mechanisms also regulate the aging program of humans. To address that, the activity of the IGF-1 signaling pathway was examined in centenarians (humans who lived more than a century) of different ethnicities. In a seminal study, Suh and colleagues identified mutations in the IGF-1 receptor that are correlated with decreased IGF-1 signaling to be more abundant among Jewish Ashkenazi centenarians compared to control individuals, members of families that do not exhibit extreme longevity.^33^ Similarly, mutations which hyper-activate FOXO3a (the DAF-16 mammalian orthologue) have been found to be linked with extreme longevity in two centenarian groups of distinct ethnicities, Japanese-Hawaiian^34^ and German.^35^ IRS2 variants were also reported to correlate with extreme longevity in an Italian subpopulation.^36^ Together, these studies strongly suggest that the aging-regulating mechanisms downstream of the IIS are conserved from worms to humans.

## SLOWING AGING PROTECTS MODEL ORGANISMS FROM NEURODEGENERATION-LINKED PROTEOTOXICITY

The developments in the research of aging and the molecular tools that enable us to alter the aging program of invertebrates and mammals opened the way to address the question of whether aging-associated processes allow protein aggregation to become toxic and initiate neurodegeneration late in life. Several proteotoxicity models have been developed in *C. elegans*, and toxicity assays have been established. If the development of conformational diseases was an aging-independent progressive process, it was expected that slowing aging will show no effect on the rate of proteotoxicity over time. However, if an aging-associated decline in the activity of protective mechanisms exposes the aged organism to proteotoxicity it was anticipated that the alteration of aging protects from proteotoxicity.

To address this question, the Morimoto laboratory created a series of transgenic worm strains each expressing a fluorescently tagged, HD-associated polyglutamine (polyQ) stretch of different lengths.^37^ They found that at least 40 glutamine repeats were required for efficient aggregation in young (day 2 of adulthood) worms. Interestingly, the threshold number of repeats needed for efficient aggregation declined with age. In worms expressing the polyQ35-YFP chimera, aggregates were visible by day 4 of adulthood, while polyQ29-YFP aggregates were not detectable earlier than day 9 of adulthood. These findings linked the polyQ aggregation to the animal’s chronological age. The direct link between the aging process and polyQ aggregation has been shown by the discovery that RNAi-mediated IIS reduction protected worm embryos from polyQ82-YFP aggregation in a DAF-16-dependent manner. IIS reduction also mitigated the toxic effect of polyQ aggregation as measured by following the worms’ motility.^37^ This study indicated that slowing aging by reducing the activity of the IIS can alleviate the toxic effects of protein aggregation and suggested that aging is a key player in exposing the aged organism to proteotoxicity and disease.

In order to test whether IIS reduction can protect worms from the aggregation of neurodegeneration-linked peptides other than polyQ and to explore the underlying molecular mechanism we employed worms that express the human Aβ in their body-wall muscles (Aβ worms^38^). Aβ aggregation in the muscles of these animals leads to progressive paralysis of the worm population. Following this phenotype we found that IIS reduction by *daf-2* RNAi also protects worms from the toxic effect associated with Aβ aggregation in a DAF-16 and HSF-1-dependent manner. Surprisingly we found that these transcription factors mediate opposing activities; HSF-1 promotes disaggregation, while DAF-16 facilitates protective active aggregation.^39^

The counter-proteotoxic effects of IIS reduction were recently extended to additional aggregative-prone, neurodegeneration-linked proteins and to worm neurons. TDP-43 is an aggregative-prone protein that when carrying specific mutations is linked to the development of amyotrophic lateral sclerosis (ALS).^40^ To test the possibility that IIS reduction alleviates the toxic effects of aggregative TDP-43 that carries disease-linked mutation, Zhang and colleagues created worms that express either wild-type fluorescently-tagged TDP-43 or the disease-linked, C terminal domain of the protein under the regulation of a pan-neuronal promoter. They found that IIS reduction by *daf-2* RNAi treatment protected the worms from TDP-43-associated motility impairments in a DAF-16 and HSF-1-dependent manner.^41^ Interestingly, TDP-1, the TDP-43 orthologue in *C. elegans*, was recently identified as a DAF-16 target and found to be involved in the regulation of lifespan.^42^

Mutations in SOD-1 have been also linked to the emergence of familial ALS.^43^ Worms that express an ALS-linked mutated SOD-1 in their neurons exhibit extensive aggregation and impaired locomotion. The knock-down of *daf-2* alleviates these phenotypes and extends the worms’ health span.^44^ Utilizing a similar approach it was shown that in worms the toxicity associated with the aggregation of ataxin-3, a polyQ-containing protein whose aggregation leads to the development of the neurodegenerative Machado–Joseph disease (MJD), can be also suppressed by reducing the activity of the IIS pathway.^45^

Together these studies clearly show that IIS reduction protects worms from proteotoxicity of various aggregative, neurodegeneration-linked proteins. However, this protection is not exclusive to aging-manipulation by IIS reduction as dietary restriction was also found to mitigate the paralysis phenotype of Aβ worms in an HSF-1-dependent manner.^46^ The discoveries that slowing aging by the manipulation of two distinct regulating pathways, the IIS and dietary restriction, established the link between the aging process and proteotoxicity and raised the question of whether this linkage is conserved from worms to mammals.

## IIS REDUCTION DELAYS THE ONSET OF ALZHEIMER’S-LIKE DISEASE IN THE MOUSE

To create a neurodegeneration model whose aging program is amenable to manipulation, Killick and colleagues^47^ crossed AD-model mice with animals which exhibit reduced IIS. They used transgenic mice that harbor the AD-linked, mutated human APP gene carrying the Swedish mutation (K670N, M671L) (*Tg2576* mice) which causes early-onset AD in humans. These animals develop Aβ plaques in the brain and exhibit behavioral impairments from midlife.^48^*Tg2576* mice were crossed with mice lacking the insulin receptor substrate 2 to achieve progeny that express Aβ and have an altered aging program (strain *Tg2576/Irs2*^−/−^). Comparison of 12-month-old *Tg2576/Irs2*^−/−^ and their age-matched *Tg2576* counterparts revealed that the deletion of *Irs2* resulted in a significant reduction of the Aβ plaque burden in the animals’ brain. Interestingly, *Tg2576/Irs2*^−/−^ had significantly less aggregated Aβ, suggesting that the deletion of *Irs2* enhances Aβ disaggregation and proteolysis. Behavioral tests revealed that the *Irs2* knock-out rescued the mice from learning and memory deficits typical to *Tg2576* animals.^49^

In another study, the Schubert group^50^ adopted an analogous approach and crossed *Tg2576* mice with either: *Irs2*^−/−^ mice, mice which lack the IGF-1 receptor exclusively in neurons (*nIgf1r*^−/−^), or with mice lacking the insulin receptor in their neurons (*nIR*^−/−^*)*. The abolishment of IRS2 rescued *Tg2576/Irs2*^−/−^ females, but not males, from premature death typical to *Tg2576* animals.^50^

To investigate the effects of IGF-1 signaling in the brain on Aβ toxicity, the researchers used the mice that exhibited reduced IGF-1R levels in the hippocampus (*Tg2576/nIgf1r*^−/−^). Both *Tg2576/nIgf1r*^−/−^ males and females were protected from the premature death typical to *Tg2576* mice. Aged *Tg2576/nIgf1r*^−/−^ animals had lower amounts of both peptides, Aβ_1-40_ and Aβ_1-42_, compared to their age-matched *Tg2576* siblings. These results revealed that the abolishment of IGF-1R in the mouse brain has a remarkable counter-proteotoxic effect. Interestingly, AD mice lacking the insulin receptor in their neurons (*Tg2576/nIR*^−/−^) and *Tg2576* animals succumbed to toxicity at similar ages,^50^ suggesting that IGF-1 and not insulin signaling promotes proteotoxicity in these animals.

In order to explore the underlying mechanism which enables IIS reduction to protect mice from AD-like disease we crossed AD-model mice with a well-established long-lived mouse strain that harbors only one copy of the gene encoding the IGF-1 receptor, to obtain long-lived AD-model animals. The AD-model strain we used expresses two mutated AD-linked genes, humanized APP_swe_ (a mutated mouse APP gene that contains the human Aβ sequence) and a hyper-active human presenilin-1 (PS1) lacking its regulatory exon (PS1ΔE9). Both transgenes were driven by the mouse prion protein promoter (APP_swe_/PS1ΔE9 mice).^51^ These mice develop relatively slow, age-dependent neurodegenerative symptoms which resembles these of human AD patients. These symptoms include behavioral impairments,^52^ neuro-inflammation, and Aβ plaque formation.^51^ By crossing these AD-model animals with the long-lived, stress-resistant *Igf1r*^+/−^ mouse strain^30^ we obtained progeny of four genotypes: 1) the original AD-model mice (*APP_swe_/PS1ΔE9*), 2) litter-mates that harbor both AD transgenes but only one *Igf1r* copy and thus exhibit reduced IGF-1 signaling (*APP_swe_/PS1ΔE9/Igf1r^+/^*^−^), 3) wild-type animals (*WT*, harboring no AD-linked transgenes and exhibiting natural IGF-1 signaling), and 4) mice that lack the AD-linked transgenes but have only one copy of the IGF-1 receptor (*Igf1r*^+/−^).

Similarly to the other groups we found that IGF-1 signaling reduction protected the mice from AD-associated memory and orientation impairments, mitigated the rates of neuro-inflammation, and largely prevented neuronal and synaptic losses.^53^ Although the total Aβ quantities as well as the levels of APP processing enzymes were similar in *APP_swe_/PS1ΔE9* and in *APP_swe_/PS1ΔE9/Igf1r*^+/−^ mice, Aβ plaques were smaller in size and of higher density in AD-model mice with reduced IGF-1 signaling compared to their litter-mates which exhibited natural levels of IGF-1 signaling. Apparently, the compaction of Aβ protects the brain by sequestering highly toxic oligomers,^8^,^54^ packing them in large fibrils of lower toxicity.^53^

Collectively, these mouse-based studies indicate that the manipulation of aging by IIS reduction protects the mammalian brain from toxic Aβ aggregation.

## MECHANISMS OF PROTECTION DOWNSTREAM OF THE IIS

Three major cellular activities are required for aggregate detoxification and clearance: disaggregation, proteolysis, and hyper-aggregation. Our discovery that DAF-16 and HSF-1 regulate disaggregation and hyper-aggregation^39^ raises several key questions including: 1) Why do cells develop opposing detoxifying machineries? 2) What cellular components disrupt aggregates? 3) Are disaggregation and proteolysis necessarily coupled? The following model provides possible answers to these questions: in young ages cells are capable of efficiently disaggregating toxic protein aggregates to enable their rapid degradation. This is the preferred mechanism as it clears the potentially hazardous protein aggregates and recycles the amino acids. Disaggregation is regulated by HSF-1, but not by DAF-16, and can be biochemically defined.^39^ Under severe aggregation load, when the disaggregation–degradation mechanism reaches its maximal capacity and cannot preserve proteostasis, a secondary detoxification mechanism is activated. This mechanism, which is controlled by DAF-16, sequesters highly toxic aggregates to create high-molecular weight (high-MW) fibrils of lower toxicity. The idea that hyper-aggregation helps protect cells from proteotoxicity is supported by discoveries that the protective chaperones HSP104^55^ and TRiC^56^ disrupt protein aggregates when they present in low concentrations but accelerate protein aggregation when concentration exceeds a certain threshold. Recently, small heat-shock proteins were reported to function in concert with HSP104 and other chaperones to empower the proteostasis network and enable disaggregation.^57^ Thus, disaggregation was predicted to function early in life and hyper-aggregation to function in late life stages. Utilizing the Aβ worm model and the paralysis assay we found that this is the case.^58^

To address the question of whether disaggregation is facilitated by a specialized mechanism, we used an *in-vitro* disaggregation assay and found that this activity is not impaired by protease or proteasome inhibitors. However, protease inhibitors prevented the degradation of Aβ, enabling its re-aggregation. This finding supports the notion that chaperones that execute disaggregation and proteases which digest the released aggregative proteins work in co-ordination to maintain proteostasis.^59^

## REDUCING IGF-1 SIGNALING AS A NOVEL COUNTER-NEURODEGENERATION STRATEGY

The insights that were obtained during the last decade suggest that the alteration of aging in general, and IIS reduction in particular, could harness the mechanisms that prevent neurodegenerative disorders from emerging in the young organism to protect the elderly from these devastating maladies. However, key questions have to be answered to assess the therapeutic potential of this approach. It was critical to test whether IIS reduction can provide protection from proteotoxicity when applied late in life. Employing the Aβ worm model and conditional *daf-2* knock-down, we discovered that late-life IIS reduction protects the worms from toxicity without affecting lifespan.^58^ Biochemical assays showed that IIS reduction late in life leads to Aβ hyper-aggregation. DAF-16 was found to play its counter-proteotoxic roles during early and late adulthood, while HSF-1 is most important during development for the protection from toxic Aβ aggregation.^58^ This temporal study showed that the counter-proteotoxic functions of IIS reduction are separable from its longevity effects, proposing that protection from toxic protein aggregation can be achieved without lifespan extension. Recently, we have found that HSF-1 also executes its longevity functions foremost during early development.^60^

**Figure 1:**
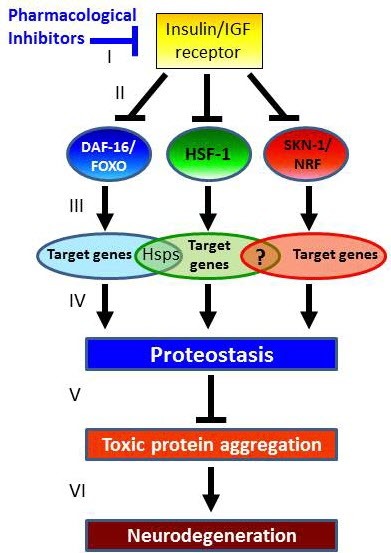
**A model for the prevention of neurodegeneration by IIS reduction.** Inhibiting the activity of the IGF-1 receptor by pharmacological agents (I) will hyper-activate the transcription factors, DAF-16/FOXO, HSF-1, and SKN-1/NRF (II). This will lead to elevated expression of the protective gene networks downstream of these transcription factors (III) and to enhanced proteostasis (IV). Functional proteostasis could efficiently disrupt protein aggregates and prevent their accumulation in cells (V). The reduced accumulation of toxic protein aggregates, a prerequisite for the development of neurodegenerative maladies, will prevent the manifestation of these disorders.

The studies described in this review point to IIS reduction as an attractive avenue towards the development of novel neurodegeneration therapies. This theme suggests that IIS reduction by pharmacological agents (Figure 1) (I), will hyper-activate the transcription factors downstream of the insulin/IGF receptor (II) and increase the expression of protective target gene networks (III). Elevated expression of these gene networks will maintain functional proteostasis (IV), prevent toxic protein aggregation from occurring (V), and prevent the manifestation of neurodegenerative disorders late in life (VI). This model calls for the development of specific IIS inhibitors and their evaluation as counter-neurodegeneration drugs. One such drug, Psammaplysene A, has been tested in cell culture and in a fly model of proteotoxicity and was found to drive FOXO3a into the neuronal nuclei and to protect motor neurons and fly eyes from toxic protein aggregation.^61^ New IIS inhibitors are being developed, and their efficiency as novel counter-neurodegeneration drugs will be tested in the years to come.

## Abbreviations:

AD: Alzheimer’s disease;
ALS: amyotrophic lateral sclerosis;
APP: amyloid precursor protein;
HD: Huntington disease;
HSF-1: heat-shock factor 1;
IGF: insulin-like growth factor;
Igf1r: insulin-like growth factor-1 receptor;
IIS: insulin/IGF signaling;
PD: Parkinson’s disease;
polyQ: polyglutamine;
PS1: presenilin-1.
